# A Proposal for a UK Ethics Council for Animal Policy: The Case for Putting Ethics Back into Policy Making

**DOI:** 10.3390/ani8060088

**Published:** 2018-06-07

**Authors:** Steven P. McCulloch, Michael J. Reiss

**Affiliations:** 1 Department of Politics and Society, University of Winchester, Winchester SO22 4NR, UK; 2 Institute of Education, University College London, London WC1H 0AL, UK; m.reiss@ucl.ac.uk

**Keywords:** Animals in Science Committee, animal rights, animal welfare ethic, Department for Environment, Food and Rural Affairs, Dutch Council on Animal Affairs, Ethics Council for Animal Policy, Farm Animal Welfare Committee, Nuffield Council on Bioethics, utilitarianism, virtue theory

## Abstract

**Simple Summary:**

Animal health and welfare policy in the UK often raises important ethical questions. Bovine tuberculosis and badger culling and the use of wild animals in circuses are good examples of controversial policy issues. In the UK, animal health and welfare advisory bodies such as the Farm Animal Welfare Committee do no not have adequate expertise to inform the moral dimensions of such policy issues. This paper proposes a body to be termed the “Ethics Council for Animal Policy” to inform the UK government on policy that significantly impacts sentient species. We review existing ethics Councils (e.g., the Nuffield Council on Bioethics and The Netherlands Council on Animal Affairs) and examine some widely used ethical frameworks (e.g., Banner’s principles and the ethical matrix). We conclude that the Ethics Council for Animal Policy should be independent of government and its members should have substantial expertise in ethics and related disciplines. A six-stage ethical framework is proposed that would help the Council to reach conclusions about such issues as whether badgers should be culled in an attempt to control bovine TB and whether wild animals should be permitted to perform in circuses.

**Abstract:**

Substantial controversy is a consistent feature of UK animal health and welfare policy. BSE, foot and mouth disease, bovine TB and badger culling, large indoor dairies, and wild animals in circuses are examples. Such policy issues are inherently normative; they include a substantial moral dimension. This paper reviews UK animal welfare advisory bodies such as the Animal Health and Welfare Board of England, the Farm Animal Welfare Committee and the Animals in Science Committee. These bodies play a key advisory role, but do not have adequate expertise in ethics to inform the moral dimension of policy. We propose an “Ethics Council for Animal Policy” to inform the UK government on policy that significantly impacts sentient species. We review existing Councils (e.g., the Nuffield Council on Bioethics and The Netherlands Council on Animal Affairs) and examine some widely used ethical frameworks (e.g., Banner’s principles and the ethical matrix). The Ethics Council for Animal Policy should be independent from government and members should have substantial expertise in ethics and related disciplines. A pluralistic six-stage ethical framework is proposed: (i) Problematisation of the policy issue, (ii) utilitarian analysis, (iii) animal rights analysis, (iv) virtue-based analysis, (v) animal welfare ethic analysis, and (vi) integrated ethical analysis. The paper concludes that an Ethics Council for Animal Policy is necessary for just and democratic policy making in all societies that use sentient nonhuman species.

## 1. Introduction

Substantial controversy is a consistent feature of animal health and welfare policy in the UK. Bovine Spongiform Encephalopathy (BSE), foot and mouth disease and *Salmonella* in eggs are notable examples. More recently, bovine tuberculosis (TB) and badger policy, large indoor dairies such as the Nocton Dairies proposal, and the use of wild animal in travelling circuses have given rise to widespread debate. These frequent controversies are despite the UK having a reputation as a leader in animal health and welfare policy. This paper focuses on animal health and welfare policy in the UK context. The paper concludes by recommending an Ethics Council for Animal Policy to inform the UK government on animal health and welfare policy.

While the focus is on the UK, the paper’s key claims are applicable to policy making in nation states more generally. Sentient animals used in farming, experimentation and for other purposes are deserving of moral status the world over. Policy that impacts sentient animals is inherently normative wherever those animals are. Thus, there is arguably a moral justification for robust ethical analysis of animal health and welfare policy on a global basis. Policy making is context-specific. Hence, ethical analysis of policy that impacts sentient animals may also be context-specific. Despite this, the key claims of this paper―that animal welfare policy is inherently normative, that this necessitates robust ethical analysis, that for political reasons this is best done independently of government―are arguably applicable universally, or at least in developed western liberal democracies.

After this introduction in Section 1, the paper is divided into five further sections. In Section 2, the case is made that animal health and welfare policy is inherently normative. This case is made based on discussing normative elements of three controversial animal health and welfare policy issues in the UK. Section 3 provides an overview of the UK animal health and welfare policy making landscape. Specifically, animal health and welfare advisory bodies are summarised and assessed with respect to their expertise in ethical analysis. Section 4 argues that UK government and its advisory bodies are not adequately constituted to inform animal health and welfare policy. It discusses two options to rectify this. First, the existing bodies could be supplemented by experts in ethics. Secondly, an additional body, an “Ethics Council for Animal Policy”, could be established. The section argues for the establishment of an Ethics Council for Animal Policy. Section 5 reviews existing ethics councils in animal welfare and similar policy areas that are characterised by substantial public controversy. In addition, the section summarises some ethical frameworks that have been used for the analysis of public policy. The purpose of the review in Section 5 is to inform the discussion in Section 6. In Section 6, the paper proposes a structure and framework for the Ethics Council that is appropriate to inform government and wider public debate on animal health and welfare policy.

## 2. The Normativity of Animal Health and Welfare Policy

Policy is concerned with a set of rules or actions of an organisation or individual. Government (or public) policy is the set of rules or actions of the executive branch of the state. Public policy therefore includes and is consistent with laws, but is a broader concept than legislation. As government policy is concerned with the distribution of resources, all public policy is necessarily normative [1,2]. In ethics, normative statements make claims about how things ought to be. Normative statements may also relate to which things are good or bad [3].

In government policy, we can distinguish between public policy for humans and animal health and welfare policy. Broadly speaking, and relative to our treatment of nonhuman sentient animals, there is considerable consensus as to how societies ought to treat human beings. Consider, for instance, the large number of signatories to the Universal Declaration of Human Rights (UDHR). In the UK context, The Human Rights Act 1998 sets out fundamental rights and freedoms that all humans in the UK are entitled to by law. They include the right to life (Article 2), freedom from torture (Article 3) and freedom from slavery and forced labour (Article 4). Thus, government policy is consistent with such laws.

In contrast, in society nonhuman animals are farmed for food, fur and fibre. They are used for experimental research in science, including medicine. Wild animals are kept in zoos for conservation, education and entertainment. Wild animals are also hunted, trapped and shot; they are culled for a variety of reasons, for instance if they are suspected of causing disease in domestic animals. The reader will notice that these uses would be prohibited should there be a similar “Animal Rights Act”. Indeed, there is significant disagreement about how animals should be treated in society. Whilst most people in the UK consume meat, dairy or fish products on a routine basis, a minority abstain on moral or other grounds. Similarly, whilst around two thirds of the British public support animal experimentation, one third are opposed [4].

These disagreements are to an extent mirrored in the philosophical literature on how human society ought to treat nonhuman animals. Singer [5] has argued for animal liberation based on equal consideration of interests, and Regan [6] has argued for abolition of animal use based on an animal rights position. In contrast, Scruton [7], for instance, has argued against any form of animal rights, based on his claim that nonhuman animals lack personhood and the relevant capacities to be rights holders. Hence, we can say that there is significant moral disagreement in the UK about how human society should treat nonhuman animals. This moral disagreement extends to mainland Europe [8], the US [9], Australia [10] and further afield.

Hence, there is a pluralism of values in society about our treatment of animals. Here, we refer to a descriptive moral pluralism about the rightful treatment of animals exists in society. We take no position on normative value pluralism, which holds that there is no single theory or framework for making ethical decisions. The proposal for an Ethics Council on Animal Policy in this paper is in part based on this descriptive moral pluralism.

Furthermore, consider the following questions concerned with animal health and welfare policy issues:

1. Should badgers be culled as part of broader policy measures to control bovine TB in cattle?
2. Should government permit year-round housing of dairy cattle?
3. Should government prohibit the use of wild animals in travelling circuses?

The following section discusses the normativity of these controversial policy issues.

### 2.1. Bovine TB and Badger Policy

Bovine TB is the most economically important animal health and welfare issue in the UK. The Department for Environment, Food and Rural Affairs (Defra) is the government department with responsibility for bovine TB policy in England. Around half of the Defra animal health and welfare budget is spent on managing the disease. Defra employs a large team of economists to inform animal health and welfare policy. However, badgers and cattle are not economic agents, so it is unclear how their interests would be considered by techniques such as cost-benefit analysis. In the case of bovine TB and badger policy, economics is a necessary part of policy making. However, it is not sufficient. The natural science evidence base [11], for instance the Randomised Badger Culling Trial (RBCT) [12], also informs badger control policy. Again, though natural science is necessary for bovine TB policy making, it is not sufficient. Should badgers be culled as part of broader policy measures to control bovine TB in cattle? The policy issue ought to be informed by the following *moral* questions, which are not exhaustive:

- Is killing a badger, as a sentient nonhuman animal, a moral harm to that individual badger?
- If killing a badger is a moral harm, how does this compare to killing a cow? Is there any moral significance in a badger being a wild animal whereas a cow is a domesticated farm animal? Does it matter, morally, that the cow has been bred to be used by humans, whereas the badger has not? What is the moral significance of the different lifespans of these species? Is there any moral significance in a far lower population of badgers compared to farmed cattle in the UK?
- Is there value in a larger badger population compared to a smaller one? If there is, is this value instrumental for the good of humans, and perhaps the environment, or does a larger badger population have intrinsic value?
- Is it morally significant that the badger is the largest land carnivore in the UK? What is the moral significance of the badger being an important part of British culture and literature, for instance Kenneth Graham’s portrayal of Mr. Badger in *The Wind in the Willows*? Morally, is it significant that the badger has traditionally been persecuted in the UK, for instance by the outlawed practice of badger baiting, and that it is a protected species in the UK (Protection of Badgers Act 1992)?
- Given there are welfare impacts to both badgers and cattle relating to bovine TB, how are these to be weighed against each other?
- For humans, how does government weigh the interests of dairy and beef farmers (who tend to support badger culling) with those of consumers or the broader public (who tend to be opposed to badger culling)?
- How are both life and killing, and positive and negative impacts related to badger policy [13], to be weighed against human impacts? Welfare impacts in badgers include suffering caused by sub-optimal (non-instantaneous) culling and those in cows include TB testing and a higher rate of slaughter in cattle. See McCulloch and Reiss [13] for further discussion.

The government’s rationale for culling badgers is ultimately an economic one. Badgers are a wildlife reservoir of disease and a significant factor in control of bovine TB in cattle. Bovine TB breakdowns cost both the government and farmers money, so it is argued that it is justifiable to cull badgers to control bovine TB in cattle [14]. Despite this, if government is to genuinely account for the interests of badgers and cattle, as well as humans, the government policy must be informed by an ethical analysis accounting for all morally relevant parties.

### 2.2. Large Indoor Dairies

Nocton Dairies is a British company that proposed an 8000 cow indoor dairy herd in Lincolnshire, the UK, in 2009. The planning application was rejected on environmental grounds. If permission for the dairy had been granted, Nocton Dairies would have been the largest dairy farm in Europe.

In the US, over 95% of lactating dairy cows are housed indoors all year [15]. In the UK, the traditional system of dairy farming is for cattle to have access to pasture during the summer months. The cows are housed during the winter months. In recent years, there has been a move to keeping some dairy cattle indoors.

High-yielding Holstein cows are genetically selected to produce high milk yields. To do this, some farms have moved to year-round housing to help manage their nutrition. Higher milk yields leads, *ceteris paribus*, to higher productivity and lower prices for supermarkets and consumers. A scientific review by the European Food Safety Authority (EFSA) recommends that cows be permitted to graze during the summer months [16]. FAWC’s opinion on housing dairy cattle indoors year round was more supportive of the more intensive system [17]. Despite the scientific evidence on the adverse welfare impact on cows of year-round housing, the UK government permits the practice. In contrast, it is mandatory for cattle to be permitted to some grazing in Sweden, Austria and Denmark.

The debate about large year-round indoor dairy herds is complex. Factors to consider include economics, rural society, the environment, food security and aesthetics, as well as animal welfare. In the indoor dairy debate, the conception of animal welfare is important. Fraser has described three conceptions of animal welfare. These are physical/functional, mental/feelings-based and naturalness. Fraser has documented how agricultural scientists and veterinary surgeons have tended to prioritise physical and functional aspects of animal welfare [18]. Indeed, in the UK, many veterinary surgeons and dairy farmers support keeping cattle indoors all year round. They point to the productivity of the cows and the physical health of the herd as an indicator of good welfare.

In contrast, other policy actors argue that the artificial indoor environment is not natural. Animal welfare organisations such as Compassion in World Farming (CIWF) [19], as well as many members of the public [20,21], support such a view. These different perspectives reveal that the concept of animal welfare is itself a normative concept. Animal welfare is analogous to human wellbeing and is concerned with what has prudential *value* for a sentient being [22]. Animal welfare is to do with what is good for an animal and therefore is itself a normative concept.

Animal welfare is a normative concept about what is of prudential value for an animal. It is also possible to investigate animal welfare scientifically. Hence, animal welfare is a normative, or value laden, concept, that can to some extent be investigated scientifically. The task of animal welfare science is to investigate what is good for an animal scientifically. Animal welfare science uses measures, or indicators, of welfare such as the time to die after being shot in badgers and lameness in dairy cattle. Animal welfare science has also developed techniques to “ask” animals what they want, such as preference testing [18].

In summary, different actors and organisations are likely to have different conceptions of animal welfare, based on normative considerations as well as differing interpretations of the empirical scientific evidence base.

### 2.3. Wild Animals in Travelling Circuses

There is a broad consensus in the UK that the welfare needs of captive wild animals cannot be met in a travelling circus environment [23,24,25,26]. (Though see Kiley-Worthington [27] who argues that the welfare needs of wild animals can be met in a travelling circus environment.) Circuses provide entertainment and are popular among some members of the public. Furthermore, those who work in circuses argue that circus life is a cultural tradition. There are only a small number of wild animals that are used in circuses. In the UK for instance, between 2013 and 2017, there were 37 wild animals registered as used in circuses [28]. The animals include six Reindeer, seven Zebra, six snakes, four Camels, four Tigers, three Raccoons, two Lions, an Ankole, a Fox, a Macaw and a Zebu. The figures compare to one billion land farm animals raised and slaughtered in the UK annually.

Much government policy on animals is justified on an implicit utilitarian basis. However, given the very small number of circus animals used in UK circuses, the benefits of entertainment for those watching circuses may outweigh any suffering of the wild animals kept for performing in them. Additionally, much animal welfare regulation is based on the principle of unnecessary suffering [29]. In “Animals, Ethics and Public Policy”, Garner has discussed how the animal welfare ethic, which describes the moral status quo, is based on the principle of unnecessary suffering. Based on the principle of unnecessary suffering, it can be argued that the use of wild animals in travelling circuses should be prohibited. Entertainment—especially that specifically related to a circus―is unnecessary and does not justify causing suffering to sentient animals [24].

Again, these considerations reveal that government policy on wild animals in circuses is a normative issue. Should government follow a more utilitarian model, which might permit the continuation of circuses? Or should it follow an unnecessary suffering model, which would likely prohibit circuses? Related to this, circus people claim that the circus is more than merely entertainment; it is a traditional way of life. Liberal democracies tend to value traditional ways of life and protect minorities from the “tyranny of the majority”. The circus debate is therefore infused with normative elements concerning the relative values of animal welfare, mass entertainment, public opinion and respect for traditional ways of life. At the time of writing in May 2018, the UK government has pledged to ban the use of wild animals in travelling circuses by 2020 [28].

## 3. A Review of the UK Animal Health and Welfare Policy Advisory Body Landscape

Section 2 has discussed the inherent normativity of animal health and welfare policy. The purpose of this section is to review advisory bodies in UK animal health and welfare policy. Given the inherent normativity of animal health and welfare policy, advisory bodies should be constituted with the expertise needed for the analysis of the moral dimensions of the policy area. In short, we should expect UK animal health and welfare advisory bodies to have significant expertise in moral philosophy, applied ethics and related disciplines. The following sub-section provides an overview of the governance of animal health and welfare policy in the UK. This is followed by an overview of key advisory bodies in Section 3.2, Section 3.3, Section 3.4, Section 3.5 and Section 3.6, including the Animal Health and Welfare Board of England (AHWBE), the Farm Animal Welfare Committee (FAWC), the Animals in Science Committee (ASC), the Companion Animal Welfare Council (CAWC) and the Wild Animal Welfare Committee (WAWC).

### 3.1. The Governance of Animal Health and Welfare Policy in the UK

Animal health and welfare policy is governed at multiple levels. Bache and Flinders characterise multilevel governance (MLG) as follows:

MLG directs attention to a complexity, cross-sectoral engagement and contestation of legitimate authority between actors organized at different territorial levels that increasingly speaks to the nature of British governance.

[30]

At the international level, the United Nations Food and Agricultural Organisation (UN FAO), the World Organisation for Animal Health (OIE), and the World Trade Organisation (WTO) influence policy. As a member state of the EU, the UK government is a signatory to Article 13 of the Treaty of Lisbon. The Treaty of Lisbon amends the Treaty on the Functioning of the EU. Article 13 recognises that animals are sentient beings and confers a duty on member states to pay full regard to animal welfare when formulating and implementing policy [31].

The UK is also subject to EU directives and regulations in animal health and welfare policy. For instance, Council Directive 1999/74/EC laying down minimum standards for the protection of laying hens and Council Regulation (EC) No 1/2005 on the protection of animals during transport and related operations. (In 2016 the British public voted in a referendum to leave the EU. The UK government has since given notice of Article 50, to begin the process of Brexit. At the time of writing in May 2018, the UK remains an EU member state, but is due to leave the EU in March 2019.)

At the national level, animal health and welfare policy is a devolved responsibility among the four nations that constitute the UK [29]. Defra has responsibility for most animal health and welfare policy in England. The Secretary of State for the Environment, Food and Rural Affairs, The Rt Hon Michael Gove (June 2017–), has overall responsibility for Defra policy. The Parliamentary Under Secretary of State for Rural Affairs and Biosecurity, Lord Gardiner of Kimble (June 2017–), has responsibility for animal health and welfare policy. The Environment, Food and Rural Affairs Committee (EFRAComm) is a Parliamentary Select Committee that scrutinises Defra policy. The All-Party Parliamentary Group for Animal Welfare (APGAW) is a Parliamentary body that keeps Members of Parliament and Peers informed about animal welfare issues and informs the development of legislation by various lobbying means [32]. At the regional level, local authorities are involved with the implementation and enforcement of policy.

In Defra, the Animal and Plant Health Agency (APHA) has responsibility for animal health and welfare. Animal health and welfare policy making in Defra is dominated by scientists and economists. In recent years, Defra has employed a smaller number of social scientists, to help with informing issues such as behaviour change related to the implementation of policy. The authors are not aware of any significant expertise in ethics or related disciplines in Defra. The first author is aware of an unpublished ethical analysis of bovine TB and badger culling conducted by a Defra civil servant some years ago. Both authors were consulted by Defra to inform the Wild Animals in Circuses Bill 2012–2013; the reason was that the Bill proposed an ethics-based ban on wild animals in travelling circuses. Aside from these, the authors are not aware of any substantive published ethical analyses on animal health and welfare policy issues conducted by Defra.

The Home Office is a separate UK government department. It is responsible for immigration, security and law and order. The Home Office has responsibility for animal health and welfare for animals involved in scientific procedures (i.e., animal experiments) under the Animals Scientific Procedures Act 1986. It discharges this responsibility principally through the Animals in Science Committee (ASC), discussed in Section 3.4.

### 3.2. Animal Health and Welfare Board of England

The AHWBE is responsible for giving strategic advice to government on animal health and welfare policy [33]. It provides oversight of the implementation of policy in England, and takes account of public health considerations. Hence, the AHWBE is not solely concerned with animal health and welfare. It is the principal source of advice to Defra ministers on all strategic animal health and welfare matters relating to kept (i.e., not wild living) animals in England. The AHWBE is formed by a Chairman, seven non-executive members and three executive members. At the time of writing (May 2018), the Chairman is Michael Seals, Chairman of the National Fallen Stock Company, an industry body. The non-executive members are mostly industry representatives and veterinary professionals. The executive members are the Chief Veterinary Officer (CVO), the Chief Executive of the APHA, and the Director of Animal Health and Welfare in Defra.

### 3.3. Farm Animal Welfare Committee

The Farm Animal Welfare Committee (FAWC) is an expert advisory committee within Defra [34]. It was established when the independent Farm Animal Welfare Council (FAWC) was disbanded in 2011. Its terms of reference are to advise Defra and the devolved administrations on the welfare of farm animals, and on any legislative or other changes to improve farm animal welfare. It provides independent scientific advice with respect to the EC Directive on the Protection of Animals at the Time of Killing. FAWC publishes all of its formal reports.

FAWC is composed of a Chair and around 13 members, one of whom generally has specialist expertise in ethics. At the time of writing (May 2018) the Chair of FAWC is Peter Jinman, a veterinary surgeon. The ethicist is David Grumett. The second author was the ethicist on FAWC (2004–12). Other members include veterinary surgeons, individuals from various sectors of the farming industry and animal welfare scientists. The FAWC *Farm Animal Welfare in Great Britain: Past, Present and Future* report includes a short section on ethics. The report proposed that the British government act as “guardian” of farm animals. It recommends that government policy should be that all farm animals in Britain have a life worth living, and an increasing number have a good life [35].

### 3.4. Animals in Science Committee

The ASC is an advisory non-departmental public body sponsored by the Home Office [36]. It includes senior academics involved in research in public and private institutions and representatives from animal welfare and animal rights NGOs. The ASC advises the Secretary of State (Home Office) on all matters concerning animals in scientific procedures. It advises welfare bodies on sharing best practice in the UK and exchanges information in the EU to coordinate best practice. The ASC operates under the Animals Scientific Procedures Act 1986. The Act permits experimental procedures to be conducted on animals based on a cost-benefit analysis. Hence, the Animal Scientific Procedures Act permits experiments on animals based on a utilitarian justification. Experiments are permitted if the estimated benefits outweigh the estimated harms. The second author was the specialist advisor to the House of Lords Select Committee on Animals in Scientific Procedures (2001–02).

### 3.5. Companion Animal Welfare Council

The CAWC was established in 1999 and was disbanded in 2017. It was an independent advisory body funded through the Welfare Fund for Companion Animals. In 2005 CAWC also received a £75,000 grant from Defra to fund activities for three years [37]. The CAWC Chair was Lord Soulsby of Swaffham Prior (1926–2017), a veterinary surgeon who sat in the House of Lords, the upper chamber of the Houses of Parliament in Westminster. CAWC had a further 20 members and provided advice on the welfare of companion animals and published its findings. It investigated and reported on animal welfare issues in companion animals and made policy recommendations to government [38]. CAWC had at least one member with specialist expertise in ethics prior to its disbanding. (James Yeates is a veterinary surgeon who has specialised in veterinary ethics, and was later appointed Chief Veterinary Officer at the Royal Society for the Prevention of Cruelty to Animals.)

### 3.6. Wild Animal Welfare Committee

The WAWC was established in 2014 to provide independent advice on the welfare of free-living wild animals in the UK. It was established with the aim of reducing harm to wild animals caused by human activity. It is composed of a Chair and around eight members, who have expertise in ecology, conservation, veterinary medicine, zoology, animal protection and criminology. The Chair at the time of writing (May 2018) is Pete Goddard, a veterinary surgeon with an interest in the welfare of wild animals. WAWC is concerned both with wild animals in general as well as the welfare of individual wild animals.

The reference to individual animals is important from a moral point of view. Many wildlife organisations, such as the Worldwide Fund for Nature (WWF), are concerned principally with conservation, i.e., protecting wildlife species. Animal welfare is grounded in moral individualism whereas conservation of species is grounded in moral holism. The issue is relevant, for instance, in the bovine TB and badger culling debate. Those that support culling often refer to the badger, *Meles meles*, not being a threatened species in the UK, despite its protected status. It is likely that many who oppose culling do so based on moral individualism, i.e., concern for individual badgers.

WAWC commissions and publishes independent reports on wild animal welfare issues that cause public and political concern. It engages with other organisations to inform its evidence-based reports [39].

## 4. A Proposal for a UK Ethics Council for Animal Policy

Section 2 made a case for the inherent normativity of animal health and welfare policy. Section 3 reviewed the animal health and welfare policy making landscape in the UK. It focused in particular on animal health and welfare advisory bodies. Given the inherent normativity of animal health and welfare policy, central government, or advisory bodies, might be expected to have the expertise to inform policy in this context.

Arguably, current UK animal health and welfare policy making is not equipped to inform the moral dimension of the policy area. There are no experts in this area on the AHWBE, which provides strategic input to policy. FAWC recognises the importance of normativity in animal health and welfare policy by its longstanding appointment of an ethicist. Despite this, FAWC has not published a report specifically on the ethics of farm animal welfare issues. FAWC’s *Farm Animal Welfare in Britain* [35], proposing government guardianship and recommending that all farm animals have a life worth living, is the publication most focused on the ethical aspect of farm animal policy. Furthermore, FAWC as a Council was abolished and FAWC as an expert advisory committee in Defra replaced it. Advice on the ethics of animal health and welfare policy, as opposed to the science or economics, is particularly susceptible to political interference. Furthermore, sentient animals, unlike humans, cannot directly represent themselves in the policy process. This contrasts with other historically oppressed groups, such as women, racial minorities and the disabled. For these reasons, the ethical analysis of animal health and welfare policy is best conducted by an independent expert body that is less susceptible to political interference.

With respect to companion animals, CAWC did appoint ethicists but the body was disbanded in 2017. This means that there is currently no specific independent advisory body informing policy on around 57 million household pets kept in the UK (Pet Food Manufacturing Association (PFMA), 2016). Similarly, despite the existence of WAWC, the body does not have the official advisory body status that FAWC does, and is not unofficially recognised by Defra as such, as CAWC arguably was. The disbandment of CAWC suggests funding problems for animal health and advisory bodies.

Furthermore, the Farm Animal Welfare Council was disbanded in 2011 and replaced with the Farm Animal Welfare Committee in the “bonfire of the quangos”. This was a response of the Conservative and Liberal Democrat Coalition Government to reduce expenditure as a result of the economic recession. The lack of recognition of WAWC may suggest it is seen as too progressive on animal welfare issues to be the default advisory body to government on wild animal welfare issues.

Hence, expert advisory input in the animal health and welfare policy area can be described as patchy, and this refers to the empirical, science-based advice alone. As this paper shows, expert advisory input on the moral dimension of animal health and welfare policy is practically non-existent.

In order to provide expert advice on the normative elements of animal health and welfare policy, there are therefore two options. The first is to strengthen the existing bodies with expertise in ethics. The second option is to establish an entirely new body for the task. Strengthening the existing bodies with additional expertise in ethics would be preferable to the status quo. However, there are major problems with this approach. These include the location of FAWC as an expert advisory committee within Defra, the lack of an advisory body on companion animal welfare issues, and the lack of official recognition of WAWC. For these reasons, we argue that it is preferable to establish a new body to inform in particular the moral dimension of animal health and welfare policy. An additional justification for a new body is as follows. The existing bodies play an important role focusing on the empirical evidence based in animal health and welfare policy. Given the necessity of such an empirical evidence base, as well as the necessity of expert ethical input, it seems sensible to establish an independent body to focus its expertise on the moral dimension.

The establishment of a UK Ethics Council for Animal Policy would mean that the government receives overlapping advice to some extent. For instance, ASC would continue to advise the Home Office on animal experimentation, and FAWC would continue to advise Defra on farm animal welfare issues. The UK Ethics Council for Animal Policy would provide further input in these areas. In this context, there are two points to make. First, the UK Ethics Council is envisaged as a body of expertise in ethics. Thus, it would conduct ethical analysis and provide expert advice on the moral dimensions of animal health and welfare policy. Secondly, government receives overlapping advice from multiple actors and organisations in many policy areas. For instance, in the policy area of human healthcare, it will receive advice from the General Medical Council and the Nuffield Council on Bioethics.

In order to inform more what the Ethics Council for Animal Policy should be and how it should operate, Section 5 reviews some existing bodies and frameworks that are relevant to the discussion.

## 5. Ethics Councils and Frameworks Used in Public Policy

In considering the structure and operation of an Ethics Council for Animal Policy, it is instructive to review similar bodies in the UK and elsewhere and the moral frameworks that are used. The current UK animal health and welfare advisory bodies were reviewed in Section 3. They were found to have shortcomings with respect to their ability to provide robust analyses of the moral dimensions of animal health and welfare policy. This section reviews some advisory bodies that are focused on ethics, as well as the moral frameworks that they use. Additionally, we review some ethical frameworks that have been developed by academics to facilitate ethical analysis of controversial policy issues. We review the following in this section and justify our approach below:

- The Nuffield Council on Bioethics
- Netherlands approach
- Pragmatic approaches (including Wolff and Sandøe and Christiansen)
- Banner’s Principles
- The ethical matrix
- Delphi techniques (including the ethical Delphi and policy Delphi)
- Public participation and deliberative democracy.

The Nuffield Council is reviewed because it is a UK-based advisory body that is internationally respected and explicitly concerned with ethical analysis of healthcare issues. Indeed, the existence of the Nuffield Council on Bioethics arguably supports the need for a similar advisory body to inform the government and public on the moral dimensions of animal health and welfare policy. The Nuffield Council exists because of moral issues that arise in modern medicine such as xenotransplantation, end of life care and euthanasia. These are moral issues that demand analysis by experts in ethics, *inter alia*, to inform policy makers in this context. In the same way, animal health and welfare issues are inherently normative, as discussed in Section 2, so, *mutatis mutandis*, the policy area also demands robust ethical analysis.

As far as the authors are aware, The Netherlands has the most developed system of governance in terms of accounting for the moral dimensions of animal health and welfare policy. For this reason, what we have termed the “Netherlands approach” is discussed here. We then examine approaches and frameworks that could be used by an Ethics Council for Animal Policy. The political philosopher Jonathan Wolff has been a member of various government advisory committees that provide advice on controversial moral issues. We review his approach here because Wolff has discussed his recommendations for how such bodies should approach controversial issues. Peter Sandøe and Stine Christiansen are reviewed as a further pragmatic approach using a mixed moral framework. Banner’s principles are reviewed because they were formulated by Michael Banner specifically for an animal welfare policy area, animal experimentation. Furthermore, FAWC has stated it follows Banner’s principles; thus, they have been applied to farm animal welfare also.

The ethical matrix and the ethical Delphi and policy Delphi have been used for the analysis of environmental, food biotechnology, and animal welfare policy issues. Hence, they are reviewed here. Finally, we briefly consider public participatory methods and deliberative democracy. These approaches are distinct from but complementary to ethics advisory bodies and frameworks. Some ethics councils, such as the Nuffield Council, conduct public participatory techniques, to inform their analyses. Furthermore, Garner [40] has recommended deliberative democracy to inform public policy on animals.

### 5.1. The Nuffield Council on Bioethics

The Nuffield Council on Bioethics in the UK is an independent body, established in 1991. The Nuffield Council has produced reports on ethical issues in biology and medicine, such as critical care decisions in fetal and neonatal medicine (2006), biofuels (2011) and emerging biotechnologies (2012). It has also produced a report on the ethics of research involving animals (2005) [41]. Chan and Harris describe the Council as “a liberal democratic, secular body that attempts to span both theoretical ethics and practical policy” [42].

Chan and Harris have reviewed the ethical frameworks the Nuffield Council has used in its reports. They found that some reports explicitly stated ethical frameworks whilst frameworks were less obvious in others. There is no defined “Nuffield Council Ethical Framework for Bioethics” but the authors found several principles that were used across the reports. These are listed below [42]:

- Avoidance of causing harm
- Prevention of harm
- Duty of beneficence
- Respect for persons and autonomy
- Justice and just resource allocation
- Informed consent
- Confidentiality and privacy
- Moral status: to whom do these principles apply (scope)?

In addition, the principles of respect for dignity and naturalness were used, but less often. Chan and Harris noted that the Council’s reports have evolved from making predominantly policy-based recommendations to a more detailed consideration of underlying ethical principles [42]. They argue that the Council should make more explicit use of ethical frameworks, because they “lend both added authority and greater clarity to the reports” [42].

In contrast to the veterinarian-, scientist- and industry-dominated FAWC, the Nuffield Council is formed by a relatively even mix of scientists, philosophers and ethicists, legal scholars and social scientists [43]. The Nuffield Council is widely respected and has been emulated in other parts of the world. In the context of this paper, there is much to learn from the Council.

The membership of the Nuffield Council is well suited to its role of ethical analysis. The medical experts and natural scientists on the Council can provide the necessary empirical information to inform ethical analysis. The Council conducts in-depth inquiries that typically take 18–24 months per report. It also responds to developments in the field through more rapid activities such as producing briefing papers, providing media interviews and writing opinion articles for the Council’s blog [44]. The Council is external to government, which allows it to be independent from political interference. The reports and background information on working procedures are published online which improves transparency.

The Nuffield Council on Bioethics is a good starting point to inform the structures and procedures of the UK Ethics Council for Animal Policy for the reasons discussed above. The Nuffield Council is focused on applied ethics, as the UK Ethics Council for Animal Policy will be. Furthermore, they are both UK advisory bodies. The next section discusses The Netherlands approach to the inherent normativity of animal health and welfare policy.

### 5.2. Netherlands Approach

The Dutch Animals Act 2011 recognises sentient animals as having intrinsic value. The Dutch Ministry of Economic Affairs, Agriculture and Innovation has used a process of ethical deliberation on policy that impacts on animals’ interests. This process was motivated by the announcement from the Ministry of Agriculture that “in future, decision-making on the treatment of animals would be as transparent as possible, indicating the considerations leading to the decision and the overriding interest or interests involved” [45]. The Dutch Animals Act and the ministerial announcement lead to direct and explicit consideration of animals’ interests in government policy making:

The interests of the animal are weighed against other interests that commonly arise in relation to the treatment of animals, such as public health, production and economics, the environment, fair trade, companionship, sport, play, enjoyment and biodiversity [45].

To fulfil the ministerial statement, the Ministry commissioned Utrecht University to create a process of ethical analysis to inform government policy making. The “Utrecht Plan” is based on a deliberative process employing the triangular interaction of intuitions/emotions, principles/moral values and facts. Deliberation using these three inputs leads to a moral judgement to inform policy making. The Dutch Ministry also considered the Nijmegen method, the dilemma method and the ethical matrix but ultimately favoured the Utrecht Plan [45].

Ethicists from Utrecht University held workshops in the Dutch Ministry and over 100 policy officials are familiar with the process [45]. The Utrecht ethicists trained two ministry policy officials as facilitators, who use the process with stakeholder participation to inform policy. The Utrecht stepped plan is outlined below [45]:

*Phase I*—*Exploration/clarification of the policy problem*
  Step 1: The dossier holder gives a short description of the policy problem and the context
  Step 2: What initial response does this case evoke in those present?
  Step 3: What else is known? What facts are missing?
*Phase II*—*Analysis of the moral dimension of the policy problem*
  Step 4: What is the moral question?
  Step 5: Who is involved in the moral question, and what are the arguments supporting their answer to the moral question?
  Step 6: Specify the ethical dilemma: what conflicting values are there?
*Phase III*—*Weighing up the arguments/values*
  Step 7: What weight is given to the arguments raised in Step 5?
  Step 8: Which course of action is preferred on the basis of these deliberations?
*Phase IV*—*Approach to the policy problem*
  Step 9: What concrete steps follow from the process?

The purpose of deliberation of ethical issues is to inform, not determine, policy-making. Ultimately, it is the minister or Secretary of State who decides policy [45].

The Netherlands Council on Animal Affairs is an independent body of experts that provides the Minister of Agriculture, Nature and Food Quality advice on multidisciplinary issues in animal welfare and health [46]. It includes around forty members, who provide advice in a personal capacity, with different types of expertise. The Council on Animal Affairs provides advice on farm, companion, equine, zoo and wild animals. Members have expertise in a wide range of areas including veterinary medicine, animal welfare science and various animal sectors (e.g., farm, experimental, companion). The Council includes an ethicist, a professor of public understanding of science and an expert in consumer behaviour. The ethicist at the time of writing (May 2018) is Franck Meijboom of the University of Utrecht, whose work focuses on animal health and welfare policy. The ethical approach used by the Council is similar to that described above used at the Ministry. Compared to the UK advisory bodies, The Netherlands Council’s work is more infused with moral considerations about animal use. Key reports in this context include *Moral issues and public policy on animals* (2010) [47] and *One health: A policy assessment framework* (2015) [48].

The Netherlands approach has several advantages. First, Dutch legislation explicitly recognises that sentient animals have intrinsic value. This intrinsic value means that the interests of the animals are considered directly, i.e., for their own sakes. Secondly, the ethical reflection process is conducted for all policy which significantly impacts on animals. Thirdly, the incorporation of stakeholders’ interests informs the deliberative process. Fourthly, the Utrecht ethicists have trained two civil servants as facilitators and a large number of policy officials are familiar with the process. Finally, the Utrecht stepped plan is suitable for a complex policy area, incorporating consideration of values, ethical theory and empirical facts.

Weaknesses of the Dutch process include the following: First, the importance attributed to the process will be influenced by the level of interest of the minister and broader political factors. Hence, the strengths of the method may be susceptible to the whim of those with authority in the department. Indeed, a civil servant has informed the first author that the success of the method depends on the interest of decision makers. Secondly, if conducted in central government, the ethical analysis will be influenced by a whole range of political factors, such as the ideologies of the governing coalition parties, the preferred policies of government, political trading in coalition governments, the stage of the electoral cycle, and so on. Some of these problems can be avoided, or at least reduced, if the ethical analysis is conducted at arm’s length to government. Indeed, the work of The Netherlands Council on Animal Affairs mitigates, as an independent body, the risk of political interference. Thirdly, although policy officials have been formally trained in the process and continue their training in the form of attending conferences on animal welfare and ethics, analysis in such complex policy areas might be better undertaken by a larger number of expert ethicists.

### 5.3. Pragmatic Approaches

#### 5.3.1. Wolffian Pragmatism

The philosopher Jonathan Wolff has discussed the contribution that ethics can make to public policy. In *Ethics and Public Policy: A Philosophical Enquiry*, Wolff argues for a pragmatic approach to policy making. Rather than a “top-down”, theory-driven approach, Wolff advocates a “bottom-up”, problem-driven approach [49]. Based on his experience as a member of the Nuffield Council Working Party on Research Involving Animals, Wolff writes:

Approaching a problem in public policy by means of the methodology “first choose your theory” could lead to interesting philosophical consequences but is very unlikely to lead to a usable contribution to current policy debates.

[49]

Wolff’s pragmatic claims about the limitations of foundational principles and moral frameworks to assess public policy are instructive. As Wolff states, policy is path-dependent and one must understand how and why the status quo is as it is [49]. However, as Wolff himself admits, philosophical input is a “vital part of the debate” [49]. Indeed, as discussed earlier, the Nuffield Council uses moral frameworks in its work, which seems unavoidable for rigorous and transparent ethical analysis. Furthermore, in their review, Chan and Harris [42] recommended greater use of explicit ethical frameworks to increase the transparency of Nuffield Council analyses.

#### 5.3.2. Sandøe and Christiansen’s Mixed Ethical Approach

Sandøe and Christiansen have assessed controversial issues that arise in public policy on animals in the light of five ethical approaches. These are contractarianism, utilitarianism, animal rights, relational views and respect for nature [8]. Sandøe and Christiansen have applied these approaches to the use of animals in food production, the control of animals with infectious diseases and the management of wild animals. Wright et al. have applied the same mixed approach to the prevention and control of highly pathogenic avian influenza (HPAI) H5N1 [50].

The motivation to use a range of ethical frameworks arises from a pluralism of views about the treatment of animals in society. In addition, as Sandøe and Christiansen write in their Introduction, “in reality, very few people, if any, stick strictly to a single, defined ethical principle” [8]. Therefore, there is a need to evaluate policy issues using a number of ethical approaches.

For the reasons of value pluralism in society and also the inherent strengths and weaknesses of the various ethical approaches, mixed ethical approaches are beneficial. The difficulty is to select the most appropriate approaches for the analysis and to adjudicate between them when their recommendations differ. Haynes has criticised Sandøe’s mixed approach on the grounds that it fails to consider hybrid views such as those of Sapontzis and DeGrazia. Haynes’ concern is that the mixed approaches Sandøe puts forward are used to justify “reformist” as opposed to more radical changes to public policy [22]. Sandøe, as a utilitarian, does not in fact hold a mixed-theory view of ethics [8]. Rather, it appears the mixed approaches are proposed first for their utility in controversial issues, secondly for educational purposes and thirdly to try to achieve agreement.

Sandøe is therefore using a mixed approach for pragmatic purposes. He recognises value pluralism in society generally, and indeed at a descriptive level also in individuals. This is despite being a utilitarian himself, in which right action is determined by a single principle, the principle of utility, for example pleasure, happiness, wellbeing or welfare. As Sandøe and Christiansen propose a mixed ethical framework to reflect pluralism of values in society, in Section 6 we propose a mixed ethical framework in large part for the same reason.

### 5.4. Banner’s Principles

Banner’s principles were formulated to apply to emerging technologies in the breeding of farm animals [51]. FAWC accepted Banner’s principles, listed below, and proposed they be applied to livestock farming generally [35]:

1. Harms of a certain degree and kind ought under no circumstances to be inflicted on an animal.
2. Any harm to an animal, even if not absolutely impermissible, nonetheless requires justification and must be outweighed by the good which is realistically sought in so treating it.
3. Any harm which is justified by the second principle ought, however, to be minimised as far as is reasonably possible.

Banner’s first principle constitutes an absolute prohibition on severe harms to animals. Absolute prohibitions are associated with deontological, or rules based ethical theories, such as human or animal rights. The second principle stipulates a utilitarian justification whereby any harms are outweighed by good consequences. Banner’s third principle is similar to the refinement principle of Russell and Burch’s 3Rs framework—reduction, refinement and replacement—which has been extensively applied to the issue of animals in research [52].

Banner’s principles could therefore be described as a type of mixed ethical approach. Strictly speaking, the framework is a principles-based, deontological framework. Deontological frameworks prescribe right action based on rules, laws, rights, duties and principles. However, the combination of an absolute prohibition and a utilitarian justification gives it a mixed flavour. It therefore has some of the strengths discussed above of mixed ethical approaches. The framework is useful principally because it develops the utilitarian analysis used with research animals to include an absolute moral prohibition, which can be defended by more fundamental moral arguments. Despite this strength, Banner’s principles are too narrow to serve as a complete ethical framework for the assessment of public policy on animals generally, and indeed were never intended as such.

Banner’s principles are instructive for the discussion and ultimate proposals in this paper. Banner’s principles include a constraint on the maximising principle in utilitarian theory. Utilitarian theory does not respect the “separateness of persons” [53,54]. Thus, the interests of some individuals can be sacrificed for those of others, if it maximises the overall good. Banner is claiming that, in the case of certain degrees and kinds of harm, this should not be the case. Thus, Banner is applying a deontological protection to animals to limit the negative impacts of utilitarian aggregation. This points to the problem with utilitarian theory generally. Utilitarian theory not only permits the sacrificing of interests if it results in a greater good, but makes it obligatory. This is particularly a risk in animal health and welfare policy, where the important interests of sentient animals are routinely sacrificed for sometimes trivial human benefits.

### 5.5. The Ethical Matrix

Mepham has developed the ethical matrix from the notion of the “common morality” [55], which he claims is represented by the framework of principalism used in human healthcare [55,56]. The four principles widely used in the West to assess medical ethical issues are [57]:

- Non-maleficence (no harm)
- Beneficence (provide benefit to wellbeing/welfare)
- Autonomy (self-determination)
- Justice (fairness in allocation of resources/goods between individuals).

To create the ethical matrix, Mepham combined non-maleficence and beneficence to form a single principle of wellbeing. The matrix is then used to apply the principles of wellbeing, autonomy and justice to all parties impacted by policy. In the broadest sense these are humans, sentient animals and the biotic environment. These groups can be further subdivided, for instance into producers and consumers in the human group and different species in the animal group. Table 1 presents an ethical matrix for food animal production [56].

The ethical matrix has been applied to a number of issues, including the use of bovine somatotrophin hormone in cows to increase milk production [58]. The ethical matrix can be used as a simple checklist of the impacts of various policy options or as a tool for more comprehensive deliberation of policy issues. The strengths of the matrix are that it facilitates the exploration of ethical issues through different moral frameworks. These are essentially utilitarian (wellbeing) and deontological (autonomy and justice) approaches. A weakness of the ethical matrix is that, in its current form, it does not include reference to virtue-based theory, which is a major approach in moral philosophy.

The ethical matrix is a very useful tool. It is particularly useful in the context of a deliberative meeting with stakeholders who do not necessarily have expertise in ethics. Part of its rationale is to simplify key ethical principles (e.g., the contraction of non-maleficence and beneficence to the principle of wellbeing) for such purposes. The Ethics Council we envisage, however, has a membership with significant expertise in ethics. Hence, there is a risk in the ethical matrix of using a framework that omits important considerations, such as virtue theory or the distinction between non-maleficence and beneficence, which may highlight salient moral points about a particular policy issue [59,60,61]. See, for instance, how in the case of bovine TB and badger control policy, a virtue-based analysis [59] adds significant value in terms of discussing salient moral issues to a utilitarian [60] and a deontological approach [61].

### 5.6. Delphi Techniques

The Delphi method has been used in policy making as it has the potential to account for complex multi-factorial issues. In the method, a coordinator or facilitator produces a questionnaire and sends this to a group of experts. Independently and anonymously, the experts complete the questionnaire and return it to the facilitator. The facilitator identifies common themes in the responses from the experts and produces further rounds of questionnaires to focus on areas that the experts have highlighted. The process is iterative and anonymity of responses eliminates the deference to authority which often occurs in face-to-face group situations [62].

An ethical Delphi approach has been developed and used to investigate the issue of genetically modified fish production [62]. In contrast to some of the approaches discussed above, the ethical Delphi does not, at least explicitly, make use of ethical theories. The ethical Delphi does, however, draw out ethical dimensions during the iterative questionnaire and response process [62]. Millar et al. considered the ethical Delphi to be useful when expert input is required, weighing of arguments is necessary and various parties are impacted by the policy issue. They claim that the method can identify value judgements of experts and areas of convergence and divergence of opinion, encourage moral reflection and provide grounds for rational decision-making [62].

Magalhães-Sant’Ana et al. [63] have used a policy Delphi to investigate the diversity of views about ethical challenges facing the veterinary profession in Ireland. The authors used vignette methodology in a three round policy Delphi with veterinary surgeons, veterinary nurses and inspectors as participants. The study investigates the participants’ views about the importance of issues in the context of ethical acceptability, perceived standards of practice and reputational risk. The final round of the Delphi explored possible solutions.

Delphi techniques can therefore inform decision-making on complex policy issues that impact on a range of parties. Since animal health and welfare policy is complex and impacts on a number of parties, the ethical Delphi is a useful tool to inform decision-making. However, although the method could be useful in certain contexts, it is not suitable as the principal tool of ethical reflection in animal health and welfare policy. The authors favour a transparent process for the Ethics Council for Animal Policy. Following the recommendations of Chan and Harris for the Nuffield Council [42], we agree that explicit ethical frameworks promotes transparency. Routine ethical analysis of animal health and welfare policy requires a broader and more transparent process than the Delphi method. Despite this, the ethical and policy Delphi techniques may have a place in the early stages of ethical analysis in some issues to investigate the views amongst expert stakeholders in animal health and welfare policy.

### 5.7. Public Participation and Deliberative Democracy

Consensus conferences, public juries and mini-publics provide fora where a selected panel of experts provides information to a group of lay citizens. The citizens then deliberate on the issues and make judgements about the policy issue. The purpose is to find out the informed judgements of citizens to feed into the policy process. Such participatory techniques are said to enhance democratic values and educate the public about policy issues.

Defra held a citizens panel on bovine TB policy in 2006 [64]. A related 2006 Defra public consultation found that 95.6% of the respondents were against a cull [65]. In the citizens panel, which had been shown presentations by experts on bovine TB and badgers, around half supported the cull:

When forced to come to a decision, there is marginal support for a cull in the context of the one-day workshop where participants are encouraged to reach a consensus decision. However, this level of support is reluctant, and heavily caveated. Furthermore, when expressing an individual viewpoint participants are equally split between pro-cull and anti-cull positions.

[64]

Despite this observation, it is not possible to draw quantitative conclusions from participatory techniques. Citizens panels are made up of a small sample of the population. The methodology is qualitative and the objective is in part to better understand the views of citizens when presented with expert information [64]. In this case, 47,308 responded to the public consultation [65] while only 48 participated in the citizens panel [64].

The idea of public participatory techniques speaks to democratic ideals, i.e., government policy should reflect an informed public. In the context of animal health and welfare policy, participatory approaches are important to inform the policy process concerning underlying moral values about the treatment of sentient animals. The authors support participatory approaches and deliberative democracy. Despite this, these approaches are not intended to provide prescriptive ethical analyses about public policy on animals. Furthermore, an analysis of public participatory approaches and deliberative democracy is beyond the remit of this paper. Interested readers should refer to Nielsen [66,67] for a discussion on public participation and Garner [40] for a paper on deliberative democracy in the context of public policy on animals.

## 6. The Ethics Council for Animal Policy

Ethics is a long established and complex discipline of study. Decisions of policy makers should be informed by ethical considerations. However, policy makers generally do not have expertise in ethical analysis. Furthermore, in the UK there is little moral expertise on tap in government for animal health and welfare policy. As discussed in Section 3.1, experts in Defra are overwhelmingly natural scientists and economists. Section 5 has reviewed relevant advisory bodies and frameworks relevant to animal health and welfare policy. The following subsections outline a proposed structure and procedures of the Ethics Council for Animal Policy, based in part on the earlier discussion.

### 6.1. Council Structure

Ethical analysis is best conducted external to government not least because ethical analysis at arm’s length from government is less susceptible to political interference. The reports and opinions of an independent body are more likely to be respected within the policy community and the public at large, since there will be fewer grounds for accusation of bias and towing the government line.

The expert body should be permanent, i.e., it should be a standing Council. The permanency of a standing Council is justified both by the nature and the scope of its responsibilities. As Mahatma Gandhi said, a nation can be judged by how it treats its animals. A permanent, standing Council is a reflection of the government’s recognition of a public genuinely concerned about sentient animals. Furthermore, policy impacts on animals are not isolated and infrequent but widespread and ongoing. A standing Council is a moral requirement because of the extent of human impact on the natural world and the fact that sentient animals are used in a multitude of ways for human ends.

The Ethics Council for Animal Policy should be composed of a Chair and, we suggest, a further twenty members. The Chair would be appointed by Parliament, which is the UK legislature. The Chair, in consultation with others, would then appoint a further twenty members to the Council. A larger Council enables flexibility and reduces workload for members. It permits the Council a greater range and depth of expertise and allows it to work on more than one investigation at any one time. The Council would also be able to co-opt additional members for expertise on an ad-hoc basis.

The Chairs of existing advisory groups, such as FAWC, are, at the time of writing, appointed by Defra, i.e., government, or the UK executive branch of government. The practice of government appointing the chair threatens the independence of any advisory body. If government appoints the chair, and the chair then appoints the remaining members, then it could be argued that the government is indirectly appointing all members of the advisory body. Thus, the advisory body is not truly independent. For this reason, we argue that there should be a move toward Parliament appointing chairs of animal health and welfare advisory bodies, including the Ethics Council for Animal Policy.

The Chair and members should be suitably qualified to contribute to ethical analysis of policy that impacts animals. The Chair and most members of the Council should have substantial training and recognised expertise in ethics. Many of these members will have a background in moral philosophy and ethics, but some may be legal scholars, political theorists and social scientists. A smaller number would have expertise in relevant areas of biology, for example animal welfare, epidemiology and veterinary science. Some of these members might have roles on other advisory bodies, such as FAWC and the ASC.

The Chair and members would each serve a three-year term, renewable for a single, further three-year term. The three-year renewable term provides a compromise between the Chair and members developing expertise and applying specific expertise in the policy areas and the working of the Council becoming susceptible to factors such as the dominance of longer standing members. To maintain independence and avoid, so far as possible, any charge of bias, Council members should not have significant vested interests in human activity which impacts on animals.

The purpose of the Ethics Council is to conduct ethical analysis of policy issues. The Council can consult relevant groups and individuals with empirical expertise about various animal policies. In the UK, these might include advisory bodies (e.g., FAWC, ASC), non-governmental organisations (e.g., the Royal Society for the Prevention of Cruelty to Animals, Compassion in World Farming, the International Fund for Animal Welfare), industry groups (e.g., the National Farmers Union), independent experts, Defra, other government departments and the public. However, the Ethics Council should *not* be a multi-stakeholder forum. Rather, the Ethics Council would be an expert body of ethicists appointed to provide a rigorous ethical input to policy making.

Parliament should fund the Ethics Council on a long-term basis. Despite this additional funding from Parliament, it is possible that the Ethics Council would more than pay for itself if its recommendations were followed. BSE, foot and mouth disease and bovine TB have cost the government and taxpayer considerable sums. It is likely that a body such as the Ethics Council would have recommended against such practices as feeding cattle MBM derived from other cows, for instance. This recommendation alone might have saved the UK government £5 billion.

### 6.2. Council Procedures

The Ethics Council would analyse and report on all public policy which might significantly impact sentient animals in the UK. It would provide independent reports and opinions to government and Parliament. The Council would act independently and have the remit to deliberate and report on policy issues as it deemed necessary. Policy issues to investigate might arise for a number of reasons. For instance, media coverage might reflect public concern about the impact of policy on sentient animals. Government, the Official Opposition or Members of Parliament might suggest policy issues to investigate. The Council itself might perceive the need to provide greater ethical clarification on policy issues. These routes to analysis and the submission of reports are not intended to be exhaustive.

The Ethics Council reports would be formally submitted to government and Parliament. In addition, the Council’s procedures and outputs would be made publicly available on its official website. While the principal purpose of the Ethics Council would be to provide ethical analysis to inform the policy process, an important role would be to inform the broader public debate about how policy impacts on animals. For this reason, the publication of reports for public consumption would be essential. The general position of this paper is that there should be high quality ethical analyses of the potential impacts of government policy on sentient nonhuman animals, the processes should be transparent and the outputs should be publicly available.

As an independent body of experts, the framework of analysis would be at the discretion of the Council. However, the framework should meet certain criteria to fulfill its purpose to inform policy makers and public debate. There are two key criteria that should dictate the framework of ethical analysis. First, the analysis should be comprehensive from a moral point of view, accounting for empirical factors. The second criterion is that the reports should be accessible to both policy makers and the public. In a similar way to scientific reports, ethical analysis of policy issues may be complex. However, as far as possible the reports should be written in a manner which can be understood by non-experts in ethics. The main findings of the reports should be included in an Executive Summary preceding more detailed analysis.

### 6.3. Outline of a Six-Step Framework of Ethical Analysis

**Figure 1:**
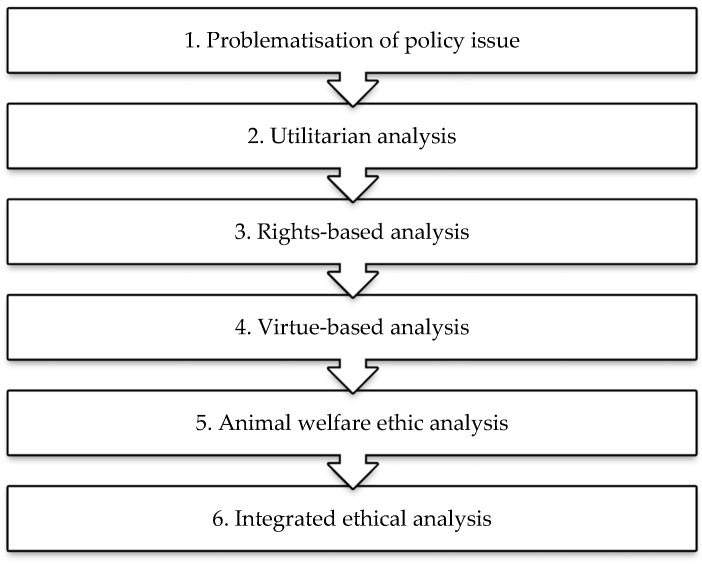
Six-stage process for ethical appraisal of policy options.

The moral framework of analysis used by the Council would reflect its expertise, the preferences of its members and suitability for particular policy issues. The moral framework discussed in this section reflects the preferences of the authors. Our preferred approach is found in Figure 1 below.

The first stage is to problematise the policy issue. In much public policy which impacts animals, the moral disagreement is at a basic level. For instance, is the *purpose* of circuses restricted to entertainment, or do circuses have scientific, educational or cultural value? Is bovine TB actually an important animal health and welfare issue, aside from its economic impacts? What is the purpose of intensifying milk production in the UK?

In the history of moral philosophy and in the modern treatment of the subject, there are four major approaches in ethics. These four approaches are utilitarianism, deontological theory, virtue theory and contract theory. The framework in Figure 1 employs three of these four major approaches: utilitarianism, a rights-based approach and virtue theory.

The fourth major moral theory is social contract theory, or contractarianism. Most contract theorists argue that since animals are not rational, they cannot take part in a contract, and so are excluded from direct moral consideration and principles of justice [54,68]. Following Rowlands [69], the authors would argue that contract theory can provide a basis for moral consideration and a theory of justice for animals. However, since major contract theorists tend to exclude animals from the sphere of justice [68], the approach is not included in the framework in Figure 1.

In utilitarian theory, the right policy is determined by a single principle, the principle of utility. This principle could be pleasure and the avoidance of pain, or happiness, wellbeing or animal welfare. Utilitarian theory is a type of consequentialism. In consequentialist theory, the right policy is determined by the consequences of the act alone. In deontological theory, the right policy is determined by a system of rules, laws, rights, duties or principles. Deontological theories are non-consequentialist. This means that the right policy might be one that doesn’t necessarily bring about the best consequences. For instance, we saw in Section 5.5 that Banner’s first principle prohibits harms to animals of a certain degree and kind. This is the case no matter what the benefits that might derive, to humans or nonhuman animals.

In virtue theory, the right policy is the one that a good (virtuous) person (or government in our case) would have. Virtues are dispositions to act in the right way through moral education and experience. The virtuous person or government, and thus right policy, would depend to some extent on the conception(s) of virtue under consideration. This would be one of many issues that would need to be deliberated by the Ethics Council. The fifth stage of the framework is the animal welfare ethic analysis. The animal welfare ethic is included because of its importance in UK animal health and welfare policy. Briefly, the animal welfare ethic holds that it is permissible for society to use animals and indeed harm them. However, according to the unnecessary suffering principle, any harms caused to animals must be necessary. Furthermore, like in Banner’s principles, harms should be minimised. The sixth and final stage is an integrated ethical analysis. The main findings are reported in an Executive Summary.

It is instructive to make two further points about the proposed moral framework. First, we have described the framework in six stages. Arguably, the problematisation stage necessarily precedes the other stages. Aside from this, the Council, or indeed any person conducting ethical analysis, would not need to follow this particular order if using this framework. We have applied stages 1–4 in a series of papers on bovine TB and badger control policy options. Utilitarian [60], animal rights [61] and virtue-based theory [59] have been applied to the policy options of (i) do nothing, (ii) badger culling, and (iii) badger vaccination. These papers provide a more comprehensive overview of utilitarian, animal rights and virtue-based theory respectively, prior to applying these theories to bovine TB and badger policy options. The science, policy and politics of the policy issue are discussed in McCulloch and Reiss [70]. The ethical analyses were empirically informed by Animal Welfare Impact Assessment of the same policy options conducted in McCulloch and Reiss [13].

We found this order of ethical analysis a natural one to follow, but as stated above it is not necessary. Indeed, within the context of the Council or any group of deliberators, there may a number of subgroups applying different ethical approaches (e.g., utilitarian, or animal rights, or virtue theory), with perhaps the Chair and certain other individuals writing up an integrated analysis in the final report.

The second point is that the findings of these approaches may conflict. For instance, consider the following illustrative example. The Council investigates the use of wild animals in travelling circuses. It concludes that a utilitarian approach finds that permitting the continuation of the use of wild animals in travelling circuses leads to the greater good. In contrast, the animal rights approach prescribes prohibiting the use of wild animal in circuses. Moral philosophers have debated such cases for millennia, and the authors would view such an outcome as reflecting the reality that different theories can arrive at different outcomes. We would also perceive this as a benefit of a mixed approach, in that using a range of leading theories is likely to better reflect the pluralism of values in a democratic multicultural society.

Related to the above point is the final stage of the integrated ethical analysis. We perceive this stage as summarising the findings of the various stages in an accessible format. In cases of disagreement, this would of course be discussed in the integrated ethical analysis. For instance, to continue with the above example, consider that the virtue-based approach and the animal welfare ethic approach are also opposed to the use of wild animals in travelling circuses. In this case, the Council’s summary might comment that four of the five approaches in the mixed framework are opposed to the policy. Indeed, the potential for conflict between ethical theories is just one part of a broader problem that is the responsibility of decision makers. Ministers may have to make policy decisions where broader factors such as economics, environmental considerations, public opinion and political factors can be in conflict. In this context, the rationale for rigorous ethical analysis, conducted by an expert Ethics Council, is to ensure that the interests of sentient animals are considered in that complex decision which political representatives must make.

## 7. Conclusions

Animal health and welfare policy in the UK and further afield is frequently characterised by substantial controversy. In the UK, BSE, foot and mouth disease and *Salmonella* in eggs have been particularly controversial. Bovine TB and badger culling, year-round indoor dairy farms and the use of wild animals in travelling circuses have similarly caused widespread debate.

Should badgers be culled as part of broader policy measures to control bovine TB in cattle? Should government permit year-round housing of dairy cattle? Should government prohibit the use of wild animals in travelling circuses? This paper argues that it is the inherent normativity of public policy that impacts animals which makes substantial controversy an ongoing feature. The questions above all include a substantial moral dimension. Despite this, there is no formal and systematic process in the UK to conduct ethical analyses of policy that impacts sentient animals.

The paper reviews the animal health and welfare policy making landscape in the UK, particularly with respect to advisory bodies. It discusses the Animal Health and Welfare Board of England (AHWBE), the Farm Animal Welfare Committee (FAWC), the Animals in Science Committee (ASC) and the Wild Animal Welfare Committee (WAWC). We find these bodies to not have the necessary expertise to adequately inform the normative element of animal health and welfare policy. Indeed, it is this lack of ethical analysis in animal health and welfare policy that is likely to contribute significantly to the controversial nature of issues such as badger culling and intensive farming.

This paper proposes a body to be termed the “Ethics Council for Animal Policy” to inform the UK government on policy that significantly impacts sentient species. To inform the structure and procedures of the Council, the paper reviews ethics councils and frameworks used to inform government policy. The paper reviews the Nuffield Council on Bioethics and what we call the “Netherlands approach”, including the Dutch Council on Animal Affairs. We then discuss ethical approaches and frameworks that have been used to inform controversial public policy areas such as Wolffian pragmatism, mixed ethical approaches, Banner’s principles, the ethical matrix, Delphi techniques, and public participation and deliberative democracy. The paper finds that none of these councils and frameworks is entirely appropriate on its own for the remit or way of working of the Ethics Council for Animal Policy. However, the review informs the development for a proposed structure and functioning of the Ethics Council.

The authors propose that the Ethics Council for Animal Policy should have a Chair and around twenty members who have significant expertise in ethics and related disciplines. The Council should use a moral framework that is sufficiently comprehensive to account for the pluralism of values found in society. The frameworks should also promote transparency to properly inform public debate about government policy on animals. The authors preferred framework is composed of six steps: (i) problematisation of the policy issue, (ii) utilitarian analysis, (iii) animal rights analysis, (iv) virtue-based analysis, (v) animal welfare ethic analysis, and (vi) integrated ethical analysis. These frames of analysis include three major moral theories, plus the key ethical framework that underpins current UK public policy on animals. Given the moral dimensions of animal health and welfare policy, an Ethics Council for Animal Policy is necessary for just and democratic policy making in all societies that use sentient nonhuman species for human ends.

## Figures and Tables

**Table 1:** An ethical matrix for food animal production (taken from Mepham [56]).

Respect for:	Wellbeing	Autonomy	Justice
Treated Organism	e.g., Animal welfare	e.g., Behavioural freedom	Intrinsic value
Producers (e.g., farmers)	Adequate income and working conditions	Freedom to adopt or not adopt	Fair treatment in trade and law
Consumers	Availability of safe food; acceptability	Consumer choice, e.g., labelling	Universal affordability of food
Biota (fauna and flora)	Protection of the biota	Maintenance of biodiversity	Sustainability of biotic populations
